# Intraprocedural Trigger Stratification via Protocolized Isoproterenol Provocation: A Mappability-Guided Strategy for Paroxysmal Atrial Fibrillation Ablation

**DOI:** 10.1155/crp/1379417

**Published:** 2025-11-29

**Authors:** Hui-yi Liu, Meng-meng Guo, Si-jia Pu, Jun-rong Jiang, Hong Yi, Hao-wei Chen, Hai-yan Zeng, Wei-dong Lin, Yu-mei Xue

**Affiliations:** ^1^Department of Cardiology, Guangdong Cardiovascular Institute, Guangdong Provincial People's Hospital (Guangdong Academy of Medical Sciences), Southern Medical University, Guangzhou, China; ^2^Medical Research Institute, Guangdong Provincial Key Laboratory of Clinical Pharmacology, Guangdong Provincial People's Hospital, Guangdong Academy of Medical Sciences, Southern Medical University, Guangzhou, China; ^3^Guangdong Provincial Geriatrics Institute, Guangdong Provincial People's Hospital, Guangdong Academy of Medical Sciences, Southern Medical University, Guangzhou, China

**Keywords:** catheter ablation, isoproterenol provocation, paroxysmal atrial fibrillation, recurrence prediction, substrate stratification, trigger mappability

## Abstract

**Objective:**

To explore the feasibility of continuous low-dose isoproterenol (ISP) in identifying atrial fibrillation (AF) triggers under conscious sedation and to investigate the association between unmappable triggers and postablation recurrence.

**Methods:**

In 50 PAF patients (Group 1), standardized ISP infusion (2–4 μg/min) was administered to provoke triggers, followed by adenosine triphosphate (ATP) challenge (30–40 mg) if no arrhythmia was induced. A matched control cohort (*n* = 96, Group 2) was selected based on baseline characteristics. Pulmonary vein isolation (PVI) was performed in all patients. Those with mappable triggers underwent additional ablation based on triggers. Additional ablation for other patient was guided by operators' discretion.

**Results:**

In Group 1, provocative testing identified mappable triggers in 35 patients (Group 1A: 34 PV triggers and 10 non-PV triggers) and unmappable triggers in 5 (Group 1B), with 10 patients showing no inducible arrhythmia (Group 1C). After 12-month follow-up, Group 1B showed significantly higher recurrence than all other groups (60.0% vs. Group 1A: 5.7%, Group 1C: 0%, and Group 2: 14.6%; *p* < 0.05).

**Conclusions:**

Continuous low-dose ISP challenge provides a pragmatic approach for intraprocedural AF trigger identification, particularly under conscious sedation. The high recurrence rate in patients with unmappable triggers underscores the imperative for advanced mapping modalities to precisely localize arrhythmogenic foci origins.

## 1. Introduction

It was well known that most of the triggers that initiate paroxysmal atrial fibrillation (PAF) originated from pulmonary veins, and pulmonary vein isolation (PVI) was the cornerstone of catheter ablation for AF [1, 2]. However, the recurrence rate for PAF after PVI remained 8%–37%, with non-PV AF triggers implicated in approximately 10%–20% of the cases [3, 4]. Provocative testing with agents such as isoproterenol (ISP), adenosine triphosphate (ATP), or rapid atrial pacing has been proposed to unmask latent triggers and guide targeted ablation. However, the clinical utility of these protocols remained debated, particularly regarding optimal drug dosing and procedural standardization.

Existing studies suggested that ISP infusion at escalating doses (5–20 μg/min) may enhance AF trigger identification [5, 6], and protocols incorporating ISP-guided ablation have demonstrated variable success rates across heterogeneous cohorts [7–9]. Importantly, high-dose ISP regimens were frequently limited by poor hemodynamic tolerance under conscious sedation [6], yet current evidence lacked consensus on dose selection criteria or objective endpoints for trigger mapping. In this study, we aimed to evaluate a standardized low-dose ISP protocol (2–4 μg/min) combined with ATP challenge, focusing on two key aspects: feasibility of sustained trigger mapping under conscious sedation, and the prognostic implications of unmappable triggers.

## 2. Methods

### 2.1. Study Population

This single-center observational study enrolled consecutive patients with drug-refractory PAF undergoing first-time catheter ablation between September 2022 and May 2023 at Guangdong Provincial People's Hospital. We prospectively enrolled all 50 patients who underwent standardized provocative testing (Group 1). Preprocedural Holter monitoring was performed in all Group 1 patients, while Group 2 controls (*n* = 96) were derived from a retrospective registry of PAF ablations during the same period, excluding those with intraprocedural drug challenge. To minimize selection bias, propensity score matching (1:2 ratio, caliper = 0.2 SD) was performed based on clinically relevant covariates—age, sex, hypertension, diabetes, stroke, PCI, and smoking status—using the SPSS 26.0 Match Cases module (IBM, Armonk NY).

Exclusion criteria were rigorously applied: structural heart disease (LVEF < 50%, severe valvular pathology, and hypertrophic cardiomyopathy); coronary artery disease (> 70% stenosis in major vessels); prior cardiac surgery/ablation; left atrial thrombus (confirmed by TEE within 48 h); and contraindications to ISP/ATP.

The study protocol (no. GDREC2019568H, September 23, 2019) received ethics approval with explicit waiver of consent for retrospective controls, while prospective participants (Group 1) provided written informed consent for provocative testing and data collection.

### 2.2. Drug Challenge and Group Definition

The drug challenge protocol comprised two sequential phases: after achieving conscious sedation, continuous ISP infusion was initiated at 2 μg/min. The rate was increased to 4 μg/min only if the heart rate remained < 120% of baseline within 10 min. The ISP infusion was discontinued either upon induction of sustained atrial arrhythmia (defined as AF/AT persisting > 30 s) or following completion of PVI. No atrial incremental pacing or programmed electrical stimulation was performed during the procedure.

For Group 1C patients with no inducible arrhythmia during the ISP phase, a bolus of ATP (30–40 mg) was administered at a dose determined by patient body weight after the PVI procedure. In patients with ISP-induced triggers (Group 1A/B), ATP was administered if either of the following occurred postablation: recurrent AF/AT post-PVI, fractionated potentials in non-PV regions. All provocations were monitored via 12-lead ECG and 3D electroanatomic mapping (CARTO 3, Biosense Webster).

### 2.3. Procedural Approach

All antiarrhythmic drugs were discontinued for ≥ 5 half-lives before ablation. Transesophageal echocardiography (TEE), pulmonary vein computed tomography (CT), or intracardiac echocardiography (ICE) was performed within 72 h before ablation to rule out left atrial thrombus. All procedures were performed under conscious sedation with fentanyl. Nonvitamin K oral anticoagulants (NOACs) were suspended once on the morning of the procedure and reapplied 4 h following the procedure.

The right femoral vein was punctured 3 times under local anesthesia, and the electrode catheter was placed into the coronary sinus through a 7F vascular sheath. The activation clotting time (ACT) was maintained at 300–350 s after two successful atrial septal punctures. Two SL1 sheaths were delivered into the left atrium. A mapping catheter (PentaRay/LASSO) and an ablation catheter (ST/SF, Thermocool SMART TOUCHSF/SF) were delivered into the left atrium for modeling, mapping, and ablation under the guidance of the CARTO 3D mapping system. Electrophysiological phenomena were recorded in detail with the multichannel electrophysiological recording system.

PVI was performed on all patients. For Group 1, non-PV ablation performed only if inducible arrhythmia sustained > 30 s post-ISP/ATP or preprocedural documented atrial flutter. Patients were classified into Group 1A if they had mappable triggers that were clearly identified (using a PentaRay, LASSO, or ablation catheter), successfully induced, and subsequently ablated to confirm a causal relationship with clinical arrhythmias. Patients with induced unmappable atrial arrhythmia were classified as Group 1B. Patients without any induced atrial arrhythmia were classified as Group 1C. All patients in Group 2 underwent PVI. Additional ablation was performed at the discretion of operators of patients in Group 1B and Group 2. Bidirectional conduction block was defined as the endpoint for linear ablation. For focal ablation, the procedure was considered successful upon the elimination or modification of the focal potential.

### 2.4. Follow-Up

Electrocardiogram (ECG) or 24-h Holter tests were performed at 1, 3, and 6 months postoperatively and every 6 months thereafter. Patients were followed up by outpatient, telephone, and WeChat consultations. The first 3 months after the procedure were defined as the blanking period. Any atrial tachycardia or atrial fibrillation (AF) lasting ≥ 30 s after the blanking period was considered a recurrence, which was the primary endpoint of this study.

### 2.5. Statistical Analysis

Continuous variables were reported as the mean ± standard deviation for normal distributions or as median for nonnormal distributions. Data were analyzed using Student's *t*-test for two-group comparisons if normally distributed or Mann–Whitney *U* test for nonparametric two-group comparisons. One-way ANOVA was employed to compare multiple sets of measurement data. Subsequently, post hoc multiple comparisons were performed to further examine pairwise differences. Specifically, when homogeneity of variances was confirmed, the LSD test was utilized for pairwise comparisons. Conversely, in cases where heterogeneity of variances was detected, Tamhane's T2 test was applied to ensure robust statistical inference. Categorical variables were presented as percentages and compared using *χ*^2^ analysis or Fisher's exact test, with Bonferroni corrections for multiple comparisons applied as appropriate. Two-tailed *p* values < 0.05 were considered statistically significant.

## 3. Results

### 3.1. Patient Characteristics

**Figure 1:**
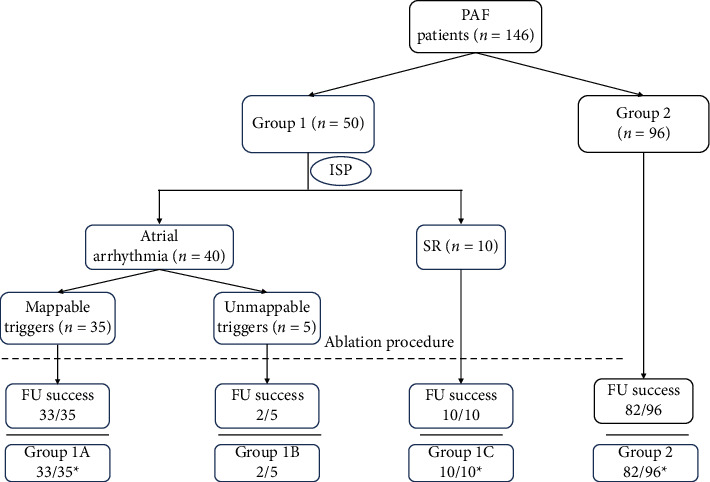
Flowchart of patients. PAF, paroxysmal atrial fibrillation; ISP, isoproterenol; FU, follow-up.

The outcome of the procedures is shown in Figure 1. A total of 146 patients who underwent RF ablation of PAF were included. Fifty PAF patients were underwent drug challenge. In 40 (80%) patients with inducible atrial arrhythmia, mappable triggers were found in 35 patients and unmappable triggers were found in 5 patients.  Table 1 summarizes the baseline characteristics of Groups 1 and 2. A drug provocation protocol was administered to 50 patients (Group 1, *n* = 50), while a propensity score-matched control cohort (Group 2, *n* = 96) was derived from PAF patients who underwent radiofrequency ablation at the same center during the same period.  Table 2 shows the baseline characteristics of Group 1A (mappable triggers, *n* = 35), 1B (unmappable triggers, *n* = 5), and 1C (noninducible, *n* = 10). There were no differences in baseline characteristics between Group 1 and Group 2. The three predefined subgroups within Group 1 also demonstrated homogeneity in baseline characteristics.

### 3.2. Trigger Provoking, Mapping, and Ablation

#### 3.2.1. AF Triggers Uncovered by Low-Dose ISP and ATP

In the pharmacologic provocation cohort (Group 1, *n* = 50), protocolized ISP infusion induced atrial arrhythmias in 80.0% (40/50) of the patients, with a mean induction time of 21.4 ± 11.4 min and cumulative ISP dose of 49.5 ± 32.0 μg. Among 55 induced arrhythmias, AF predominated (63.6%, 35/55), followed by atrial flutter (14.5%, 8/55), atrial tachycardia (7.3%, 4/55), and premature atrial contractions (14.5%, 8/55), with one case of vagally mediated PAC resolving after atropine.

**Figure 2:**
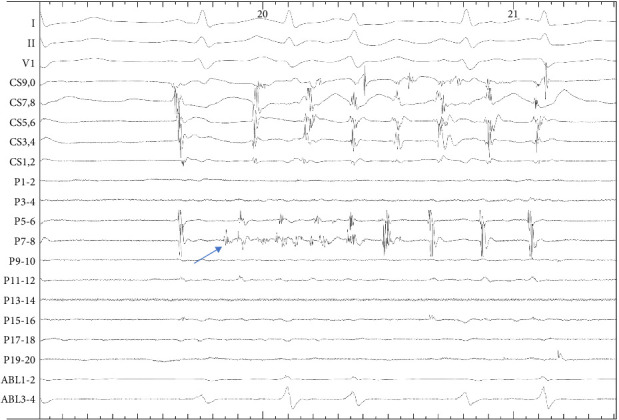
Examples of AF triggers originating from the LSPV. From top to bottom: surface ECG leads I, II, and V_1_; the coronary sinus (poles 9, 10 to 1, 2), the PentaRay catheter in the left superior pulmonary vein (poles 1, 2 to 19, 20), and the ablation catheter in the left atrium (poles 1, 2 to 3, 4).

**Figure 3:**
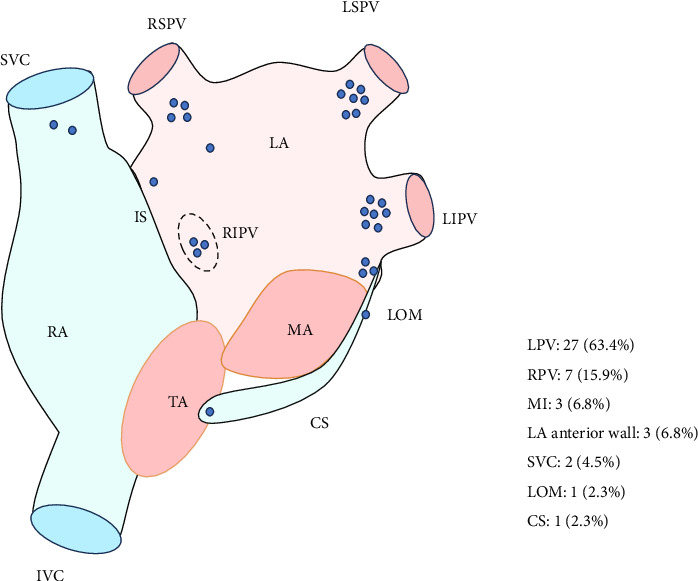
The distribution of triggers in Group 1A. LPV, left pulmonary vein; RPV, right pulmonary vein; MI, mitral isthmus; LA, left atrium; RA, right atrium; IS, interatrial septum; LOM, ligament of Marshall; CS, coronary sinus; SVC, superior vena cava; MA, mitral annular; TA, tricuspid annulus.

In 35 patients with mappable triggers (Group 1A), total 38 triggers were found and PV triggers constituted 86.8% (33/38). Among PV triggers, 78.8% (26/33) originated from LPVs and 88.6% (31/35) of the patients were affected. Non-PV triggers (13.2%, 5/38 foci) localized to mitral isthmus (*n* = 2), left atrial anterior wall (*n* = 2), and superior vena cava (*n* = 1). Postablation ATP challenge was administered to 68.6% (24/35) of Group 1A patients, inducing arrhythmias in 16.7% (4/24) with the following trigger distribution: mitral isthmus (*n* = 1), pulmonary vein (*n* = 1), superior vena cava (*n* = 1), and one multifocal origin involving the left atrial anterior wall, coronary sinus, and ligament of Marshall.  Figure 2 shows an examples of AF triggers originating from the LSPV, and the localization of triggers is summarized in Figure 3.

In Group 1B, sustained AF induced in all patients during ISP, but triggers were unmappable. After PVI and empirical ablation, two patients accepted ATP, and no atrial arrhythmia was provoked. In Group 1C, 10 patients accepted both the ISP and ATP challenge, but no atrial arrhythmia was induced. The mean dose of ATP was 35.4 ± 5.0 mg. There were zero hemodynamic or arrhythmic adverse events related to provocation protocols.

#### 3.2.2. The Characteristics of the Procedure

PVI was universally performed. All identified triggers were successfully ablated in Group 1A (mappable triggers, *n* = 35). Patients in Group 1B (unmappable triggers, *n* = 5) all received empirical substrate modification, including mitral isthmus line (5/5, 100%), left atrial roof line (3/5, 60%), ligament of Marshall (2/5, 40%), tricuspid isthmus line (1/5, 20%), LA inferior line (1/5, 20%), and LA anterior line (1/5, 20%). Nonterminable AF persisted in 3 patients (60%) and ibutilide was required for sinus rhythm restoration. Group 1C (noninducible, *n* = 10) received adjunctive ablation in 30% (3/10) guided by AFL documented on preprocedural Holter: SVC isolation plus tricuspid isthmus (*n* = 2) and coronary sinus ablation (*n* = 1).

The procedural characteristics of Group 1 and Group 2 are summarized in Table 3. Compared with Group 2, the mitral isthmus line and LOM ablation rates were higher in Group 1 (2.6% vs. 11.4%, *p* < 0.01; 0.0% vs. 12.0%, *p* < 0.01). In Group 2, 14.6% (14/96) patients underwent SVC isolation and 20.8% (20/96) received tricuspid isthmus ablation. Nineteen patients could not recover to SR and required DCCV (4/96, 4.2%) or ibulitide (15/96, 15.6%). Pericardial effusions occurred in two cases (1 in Group 1 and 1 in Group 2, *p*=1.0).

### 3.3. Outcome of Follow-Up

**Figure 4:**
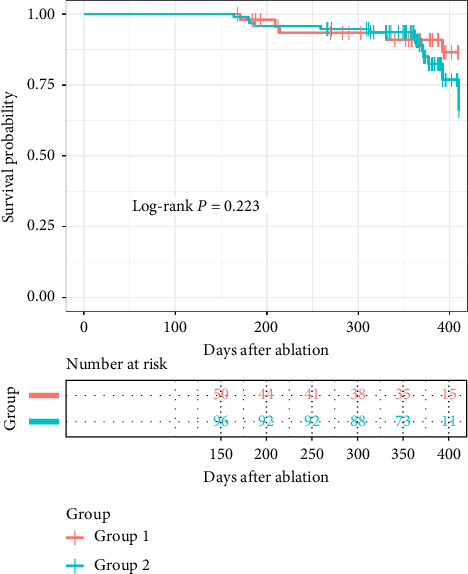
Kaplan–Meier survival analysis of freedom from arrhythmia after 1-year follow-up comparing Group 1 and Group 2 in all patients.

**Figure 5:**
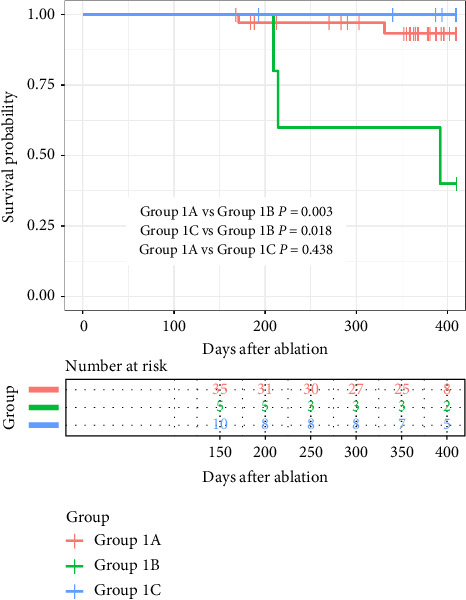
Kaplan–Meier survival analysis of freedom from arrhythmia after 1-year follow-up comparing Group 1A, 1B, and 1C in Group 1.

During the blanking period, 93.9% of the patients in Group 1 and 90.6% in Group 2 were free from early atrial arrhythmia recurrence (*p*=0.408). During a mean follow-up of 376.8 ± 125.2 and 359.0 ± 50.3 days, five patients (10.0%, AF in three patients and atrial tachycardia in two patients) in Group 1 and 14 patients (14.6%, AF in ten patients and atrial tachycardia in four patients) in Group 2 experienced atrial arrhythmia recurrence. The difference was nonsignificant between the two groups (*p*=0.435) (Table 3). However, significantly higher recurrence rates were observed in Group 1B (3/5, 60%) compared with Group 1A (2/35, 5.7%; *p* < 0.001), Group 1C (0/10, 0%; *p*=0.003), and Group 2 (14/96, 14.6%; *p*=0.033). Conversely, recurrence did not differ between Group 1A and Group 1C (5.7% vs. 0%, *p*=0.438) (Figure 4). Two patients with recurrent atrial arrhythmia in Group 1A accepted repeat ablation on days 171 and 331 after the first ablation due to PV reconnection and SVC reconnection, respectively. The Kaplan–Meier survival analysis showed no difference between Group 1 and Group 2 in freedom from atrial arrhythmia (log rank *p*=0.223) (Figure 4). Subgroup survival analysis found that the recurrence rate of Group 1A and 1C was significantly decreased when compared with Group 1B (log rank *p*=0.003 and 0.018) (Figure 5). During follow-up, there was no difference in pacemakers implanted or left atrial appendage exclusion (LAAC) between the two groups.

## 4. Discussion

### 4.1. Main Findings

The principal findings demonstrate that protocolized ISP provocation achieved an 80% rate of inducible arrhythmias in paroxysmal AF patients, with pulmonary veins constituting the predominant trigger source (86.8% of triggers). Critically, patients harboring unmappable triggers (Group 1B) exhibited higher recurrence risk compared with those with mappable substrates, underscoring the prognostic significance of trigger localization beyond mere induction.

### 4.2. Triggers and Their Identification in PAF

AF exemplifies the paradigm of Coumel's triangle, a conceptual framework established since the 1960s, which describes arrhythmogenesis as the dynamic interplay among triggers, an arrhythmogenic substrate, and modulating factors [10]. In PAF, triggers played a pivotal role, with the characteristic short-lived and self-terminating nature of the arrhythmia [11] attributable primarily to ectopic triggers in the presence of minimal or no abnormal atrial substrate.

The recurrence rate of AF after initial PVI remains high in patients with PAF, approaching 40% at 1-year follow-up [12]. This led to the evaluation of the effectiveness of trigger ablation.

AF triggers are usually represented by ectopic atrial foci. Studies confirm that triggering of PAF by premature atrial complexes (PACs) is a general rule, including when the arrhythmia occurs in patients with organic heart disease. Preprocedural ambulatory monitoring solidifies the role of PACs as the principal trigger for PAF. Study reported that a rule affirmed in 95.3% (222/233) of documented episodes in 90 PAF patients. The anatomical distribution of these triggering PACs was predominantly left atrial (74.3%), yet a significant proportion originated from the right atrium (15.3%). This spatial insight is clinically critical, as identifying a right atrial origin could prevent unnecessary left atrial ablation. These findings collectively advocate for a pathophysiology-guided ablation strategy [13].

While the PVs were the primary source of arrhythmogenic triggers in AF, other supraventricular tachycardias could also initiate this condition. Some AF originated from triggers of supraventricular tachycardia (e.g., AVNRT, AVRT, and FAT), which can be identified by intraoperative electrophysiological study. In a study on paroxysmal AF patients, up to 18.8% had inducible supraventricular tachycardia triggers. Ablation targeting only these foci yielded an excellent long-term success rate of 91.5% [14]. A 2010 study found that 10.1% of the patients diagnosed with AF and scheduled for conventional ablation actually had undiagnosed supraventricular tachycardia. After ablating only the supraventricular tachycardia, AF recurrence was 7.7%, indicating that electrophysiological study can help avoid unnecessary AF ablation and provide more precise and effective treatment [15].

Various intraoperative induction approaches, including atrial burst pacing (ABP) and drug challenge, were also employed to provoke triggers. However, studies have shown that ABP induction did not predict clinical recurrence at follow-up [16] and those induced organized atrial arrhythmias may not be clinically relevant [17]. The sensitivity and specificity of ISP for inducing AF in patients with PAF was high regardless of the clinical subtype, whether vagal, adrenergic, or occasional [6]. Crawford et al. reported that AF could be induced by ISP in 87% of PAF patients prior to ablation. The most common triggers identified by ISP were PV triggers. Non-PV triggers were identified in 15%–23% of the patients [8, 18]. Previous studies have demonstrated that AF induced by ISP infusion typically exhibit both dose-dependent characteristics. ISP infusions usually started at 5 μg/min and gradually increased to 20 μg/min. Also, AF was often inducible at a mean dose of 15 ± 5 μg/min [18, 19]. It was also recommended to use a protocol-graded infusion of ISP using up to 20–30 μg/min and AF induced for at least 10 min [20]. Though ISP has been used in various studies to assess residual AF triggers and shown to induce AF in a dose-dependent manner [6], it has not been conclusively demonstrated that ISP infusion was associated with improved outcomes following AF ablation [8, 21]. Meanwhile, high-dose ISP may result in chest discomfort, hypotension, and electrocardiographic changes suggestive of ischemia [6], making routine drug challenge during the procedure controversial.

In the present study, a low dose of ISP (2 μg/min and 4 μg/min) was chosen and continuously administrated during the procedure. A total of 80% of the patients with PAF developed atrial arrhythmia induced by ISP, which was not lower than that of the routine dose of ISP [18, 22]. Meanwhile, none of the patients experienced any adverse events related to drug challenge. Non-PV triggers were identified by ISP in 14.3% of patients, which originated from MI, LA anterior wall and SVC. The results were not lower than those obtained in other studies [2, 23, 24]. One patient developed pericardial effusion during the procedure, similar to the control group. Crucially, ISP's clinical value extended beyond induction: patients with unmappable triggers (Group 1B) exhibited 12-fold higher recurrence than mappable counterparts (*p* < 0.001). These findings mandate both ISP provocation to identify candidates for substrate modification in non-PV and unmappable groups and precision mapping such as ripple mapping for ISP-induced unmappable foci.

It was well known that ATP or adenosine induces dormant PV conduction, and additional ablation of reconnected PVs reduced postoperative recurrence rates [25]. Several case reports suggested that adenosine or ATP was more likely to identify triggers originating outside the PVs [26–28]. Studies have reported higher rates of non-PV triggers when ATP was used after PVI [21, 24, 29]. Furthermore, it has been reported that all triggers confirmed by ATP were sustained by the administration of ISP [8]. In the present study, we also found that ATP was effective in provoking non-PV triggers after infusion of ISP.

The above results consistently supported a tailored ablation approach that selectively targeted extra-PVI triggers, particularly in patients with PAF who have a long history of palpitations and no structural heart disease or cardiovascular risk factors [14, 15, 30]. This strategy, when applied to a carefully selected patient cohort, proved effective by eliminating AF-triggering arrhythmias through a simpler and faster procedure. Although the tailored method may appear time-consuming due to the need for a detailed electrophysiological study, the time invested is offset by avoiding unnecessary prolonged PVI and enhancing single-procedure success [15]. An integrated strategy—combining clinical history, analysis of recordings from Holter or implantable devices, baseline EPS, and drug challenge—enriched the clinical depiction of AF episodes and allowed proactive ablation of concomitant arrhythmias and dormant triggers. A deeper understanding of PAF pathogenesis and the advancement of technologies that guarantee short procedure times, efficacy, and safety for patients and staff alike could result in a significantly higher single-procedure success rate for AF ablation.

### 4.3. Non-PV and Unmappable Triggers of PAF

While reconnection of PVs was the predominant mechanism responsible for arrhythmia recurrence after PVI, many studies have shown that triggers outside the PV region were also important. Dukkipati et al. reported that even though permanent PVI was achieved in patients with PAF, the 1-year recurrence rate of AF was still as high as 29% [31]. Zhao et al. [32] reported that 26.6% of PAF patients with LVEF ≥ 50% had non-PV triggers, and the rate increased to 69.1% in patients with LVEF ≤ 35%. Typically, non-PV triggers were particularly prevalent in inferior left atrial triggers, SVC, CS, CT, and LAFW triggers [23, 33–35], as these sites were the anatomical and electrical connections between the muscle and the atria. Other sites of AF triggers included the persistent left superior vena cava [36] and its remnant, the ligament of Marshall [37]. Our data refined this paradigm that 80% (4/5) non-PV triggers localized to MI and LAAW. The presence of non-PV AF foci has been reported to be a significant clinical predictor of AF recurrence after ablation [3] as the presence of non-PV triggers may reflect a greater degree of atrial anatomical remodeling, which means it was difficult to map all triggers during the procedure [3].

Unmappable AF foci increased the risk of recurrence by 5.58-fold [2]. Eliminating non-PV triggers by additional ablation could affect the clinical outcome of PAF ablation [32]. In the present study, although the recurrence rate following initial ablation in the drug challenge group did not decrease, it did, among the drug challenge group, show a higher recurrence rate in patients with unmappable non-PV triggers than those with mappable triggers. These unmappable substrates signify advanced electropathology, such as autonomic dysregulation or microfibrosis, where empirical ablation demonstrates constrained efficacy. This mandates the integration of precision mapping, for instance, biatrial simultaneous mapping, to overcome localization barriers.

### 4.4. Study Limitations

There are several limitations to this study. First, as a single-center prospective cohort study, unmeasured confounders may persist despite propensity score matching. Validation through multicenter randomized trials is essential. Second, the drug provocation cohort (*n* = 50) provided adequate power for primary endpoint analysis, but subgroup analyses—particularly Group 1B (unmappable triggers, *n* = 5)—require cautious interpretation. Future studies should target ≥ 30 patients per subgroup. Third, restricted catheter positions in the left/right atria may have compromised trigger localization. Integration of high-density panoramic mapping and simultaneous biatrial acquisition would enhance spatial resolution for elusive foci. The present study focuses exclusively on the identification of intraprocedural triggers, without assessing potential modulatory factors (e.g., vagally mediated influences). Future remapping procedures in patients with recurrent AF are necessary to evaluate the clinical significance of pulmonary vein reconnection as a contributing factor.

## 5. Conclusion

Protocolized ISP provocation effectively identified mappable AF triggers during ablation procedures. Patients with unmappable triggers exhibited significantly higher recurrence rates, underscoring the clinical value of trigger localization for guiding adjunctive ablation.

## Figures and Tables

**Table 1:** Baseline characteristics of included people.

	Group 1 (*n* = 50)	Group 2 (*n* = 96)	*P*
Age	58.1 ± 13.0	57.2 ± 11.5	0.656
Male (%)	34 (68.0)	65 (67.7)	0.971
Hypertension (%)	17 (34.0)	32 (33.3)	0.935
Diabetes (%)	4 (8.0)	5 (5.2)	0.506
Stroke (%)	7 (14.0)	8 (8.3)	0.285
CHA_2_DS_2_ -VASc scores	1.42 ± 1.51	1.29 ± 1.37	0.605
Smoke (%)	10 (20.0)	15 (15.6)	0.505
Prior PCI (%)	0 (0.0)	0 (0.0)	/
LA diameter (mm)	35.5 ± 4.9	34.8 ± 3.1	0.308
LVEF (%)	64.0 ± 3.9	65.5 ± 3.9	0.678
LVDd (mm)	45.3 ± 4.1	44.9 ± 3.7	0.565
AF duration [months, M (P25, P75)]^a^	9.5 (3.6, 24.0)	12.0 (3.0, 36.0)	0.109

Abbreviations: AF, atrial fibrillation; CHA_2_DS_2_-VASc, congestive heart failure, hypertension, age ≥ 75 years, diabetes mellitus, stroke, vascular disease, age 65–74 years, sex category (female); LVEF, left ventricular ejection fraction; LVDd, left ventricular diastolic dimension; LAAC, left atrial appendage closure; LA, left atrium; PCI, percutaneous coronary intervention.

^a^Results are presented as median (interquartile range).

**Table 2:** Baseline characteristics of the drug challenge group.

	Group1A (*n* = 35)	Group1B (*n* = 5)	Group1C (*n* = 10)	*P*
Age	58.3 ± 12.9	61.0 ± 13.9	56.1 ± 14.1	0.788
Male (%)	25 (71.4)	3 (60.0)	6 (60.0)	0.334
Hypertension (%)	11 (31.4)	2 (40.0)	4 (40.0)	0.927
Diabetes (%)	2 (5.7)	0 (0.0)	2 (20.0)	0.125
Stroke (%)	6 (17.1)	0 (0.0)	1 (10.0)	0.822
CHA_2_DS_2_-VASc scores	1.4 ± 1.4	1.4 ± 1.1	1.5 ± 2.1	0.983
Smoke (%)	9 (25.7)	3 (60.0)	0 (0.0)	0.077
PCI (%)	0 (0.0)	0 (0.0)	0 (0.0)	—
LA diameter (mm)	35.9 ± 4.9	36.0 ± 1.4	33.7 ± 5.7	0.449
LVEF (%)	64.1 ± 2.8	63.5 ± 4.8	64.0 ± 5.8	0.957
LVDd (mm)	45.7 ± 3.5	44.5 ± 2.1	44.2 ± 6.0	0.561
AF duration [months, M (P25, P75)]^a^	7.2 (3.0, 12.0)	12.0 (3.0, 72.0)	24.0 (5.1, 45.0)	—

Abbreviations: AF, atrial fibrillation; CHA_2_DS_2_-VASc, congestive heart failure, hypertension, age ≥ 75 years, diabetes mellitus, stroke, vascular disease, age 65–74 years, sex category (female); LVEF, left ventricular ejection fraction; LVDd, left ventricular diastolic dimension; LAAC, left atrial appendage closure; LA, left atrium; PCI, percutaneous coronary intervention.

^a^Results are presented as median (interquartile range).

**Table 3:** Procedural characteristics and outcomes of follow-up of included patients.

	Group 1 (*n* = 50)	Group 2 (*n* = 96)	*P*
Procedural characteristics, *n* (%)			
PVI	50 (100)	96 (100)	/
SVC isolation	5 (10.0)	14 (14.6)	0.481
Tricuspid valve isthmus	8 (16.0)	20 (20.8)	0.322
Mitral isthmus	13 (26.0)	3 (3.2)	< 0.01^∗∗^
LA roof line	7 (14.0)	8 (8.3)	0.285
LA inferior line	2 (4.0)	4 (4.2)	0.962
CS ablation	3 (6.0)	1 (1.0)	0.086
LOM	6 (12.0)	0 (0.0)	< 0.01^∗∗^
DCCV	0 (0.0)	4 (4.2)	0.353
Ibutilide	3 (6.0)	15 (15.6)	0.093
Procedural complication, *n* (%)			
Cardiac tamponade	1 (2.0)	1 (1.0)	1.000
Outcome of follow-up			
Early recurrence	3 (6.1)	9 (9.4)	0.481
Late recurrence	5 (10.0)	14 (14.6)	0.435
Pacemaker implantation during follow-up (%)	1 (2.0)	0 (0.0)	0.160
LAAC (%)	1 (2.0)	0 (0.0)	0.147

Abbreviations: PVI, pulmonary vein isolation; SVC, superior vein cava; LA, left atrium; CS, coronary sinus; LOM, ligament of Marshall; DCCV, direct-current cardioversion.

^∗∗^ *p* < 0.01.

## Data Availability

The data that support the findings of this study are available from the corresponding author upon reasonable request.

## References

[1] Haïssaguerre M., Jaïs P., Shah D. C. (1998). Spontaneous Initiation of Atrial Fibrillation by Ectopic Beats Originating in the Pulmonary Veins. *New England Journal of Medicine*.

[2] Takigawa M., Takahashi A., Kuwahara T. (2015). Impact of Non-pulmonary Vein Foci on the Outcome of the Second Session of Catheter Ablation for Paroxysmal Atrial Fibrillation. *Journal of Cardiovascular Electrophysiology*.

[3] Chang H. Y., Lo L. W., Lin Y. J. (2013). Long-Term Outcome of Catheter Ablation in Patients with Atrial Fibrillation Originating from Nonpulmonary Vein Ectopy. *Journal of Cardiovascular Electrophysiology*.

[4] Lin W. S., Tai C. T., Hsieh M. H. (2003). Catheter Ablation of Paroxysmal Atrial Fibrillation Initiated by Non-pulmonary Vein Ectopy. *Circulation*.

[5] Crawford T., Chugh A., Good E. (2010). Clinical Value of Noninducibility by high-dose Isoproterenol Versus Rapid Atrial Pacing After Catheter Ablation of Paroxysmal Atrial Fibrillation. *Journal of Cardiovascular Electrophysiology*.

[6] Oral H., Crawford T., Frederick M. (2008). Inducibility of Paroxysmal Atrial Fibrillation by Isoproterenol and its Relation to the Mode of Onset of Atrial Fibrillation. *Journal of Cardiovascular Electrophysiology*.

[7] Lee K. N., Roh S. Y., Baek Y. S. (2018). Long-Term Clinical Comparison of Procedural End Points After Pulmonary Vein Isolation in Paroxysmal Atrial Fibrillation: Elimination of Nonpulmonary Vein Triggers Versus Noninducibility. *Circulation: Arrhythmia and Electrophysiology*.

[8] Sakamoto Y., Osanai H., Hiramatsu S. (2020). Efficacy of Isoproterenol in the Evaluation of Dormant Conduction and Arrhythmogenic Foci Identification in Atrial Fibrillation Ablation. *BMC Cardiovascular Disorders*.

[9] Aldaas O. M., Darden D., Mylavarapu P. S. (2022). Association of Isoproterenol Infusion During Catheter Ablation of Atrial Fibrillation with Outcomes: Insights from the UC San Diego AF Ablation Registry. *Journal of Interventional Cardiac Electrophysiology*.

[10] Coumel P. (1993). Cardiac Arrhythmias and the Autonomic Nervous System. *Journal of Cardiovascular Electrophysiology*.

[11] Hindricks G., Potpara T., Dagres N. (2021). 2020 ESC Guidelines for the Diagnosis and Management of Atrial Fibrillation Developed in Collaboration with the European Association for Cardio-Thoracic Surgery (EACTS): the Task Force for the Diagnosis and Management of Atrial Fibrillation of the European Society of Cardiology (ESC) Developed with the Special Contribution of the European Heart Rhythm Association (EHRA) of the ESC. *European Heart Journal*.

[12] Bunch T. J., Cutler M. J. (2015). Is Pulmonary Vein Isolation Still the Cornerstone in Atrial Fibrillation Ablation?. *Journal of Thoracic Disease*.

[13] Vincenti A., Brambilla R., Fumagalli M. G., Merola R., Pedretti S. (2006). Onset Mechanism of Paroxysmal Atrial Fibrillation Detected by Ambulatory Holter Monitoring. *EP Europace*.

[14] Palamà Z., Robles A. G., Paoletti M. (2023). Long-Term Follow-Up in Paroxysmal Atrial Fibrillation Patients with Documented Isolated Trigger. *Frontiers in Cardiovascular Medicine*.

[15] Sciarra L., Rebecchi M., De Ruvo E. (2010). How Many Atrial Fibrillation Ablation Candidates Have an Underlying Supraventricular Tachycardia Previously Unknown? Efficacy of Isolated Triggering Arrhythmia Ablation. *Europace*.

[16] Leong‐Sit P., Robinson M., Zado E. S. (2013). Inducibility of Atrial Fibrillation and Flutter Following Pulmonary Vein Ablation. *Journal of Cardiovascular Electrophysiology*.

[17] Huang W., Liu T., Shehata M. (2011). Inducibility of Atrial Fibrillation in the Absence of Atrial Fibrillation: what Does it Mean to be Normal?. *Heart Rhythm*.

[18] Elayi C. S., Di Biase L., Bai R. (2013). Administration of Isoproterenol and Adenosine to Guide Supplemental Ablation After Pulmonary Vein Antrum Isolation. *Journal of Cardiovascular Electrophysiology*.

[19] Tutuianu C., Pap R., Riesz T., Bencsik G., Makai A., Saghy L. (2019). Is Adenosine Useful for the Identification of Atrial Fibrillation Triggers?. *Journal of Cardiovascular Electrophysiology*.

[20] Calkins H., Hindricks G., Cappato R. (2017). 2017 HRS/EHRA/ECAS/APHRS/SOLAECE Expert Consensus Statement on Catheter and Surgical Ablation of Atrial Fibrillation. *Heart Rhythm*.

[21] Zhang J., Tang C., Zhang Y., Su X. I. (2014). Origin and Ablation of the Adenosine Triphosphate Induced Atrial Fibrillation After Circumferential Pulmonary Vein Isolation: Effects on Procedural Success Rate. *Journal of Cardiovascular Electrophysiology*.

[22] Della Rocca D. G., Di Biase L., Mohanty S. (2021). Targeting Non-pulmonary Vein Triggers in Persistent Atrial Fibrillation: Results from a Prospective, Multicentre, Observational Registry. *EP Europace*.

[23] Cheng H., Dai Y. Y., Jiang R. H. (2014). Non-Pulmonary Vein Foci Induced Before and After Pulmonary Vein Isolation in Patients Undergoing Ablation Therapy for Paroxysmal Atrial Fibrillation: Incidence and Clinical Outcome. *Journal of Zhejiang University-Science B*.

[24] Hayashi K., An Y., Nagashima M. (2015). Importance of Nonpulmonary Vein Foci in Catheter Ablation for Paroxysmal Atrial Fibrillation. *Heart Rhythm*.

[25] Macle L., Khairy P., Weerasooriya R. (2015). Adenosine-Guided Pulmonary Vein Isolation for the Treatment of Paroxysmal Atrial Fibrillation: an International, Multicentre, Randomised Superiority Trial. *The Lancet*.

[26] Miyazaki S., Takahashi Y., Fujii A., Takahashi A. (2011). Adenosine Triphosphate Exposes Multiple Extra Pulmonary Vein Foci of Atrial Fibrillation. *International Journal of Cardiology*.

[27] Hioki M., Matsuo S., Yamane T. (2012). Adenosine-Induced Atrial Tachycardia and Multiple Foci Initiating Atrial Fibrillation Eliminated by Catheter Ablation Using a Non-contact Mapping System. *Heart and Vessels*.

[28] Ip J. E., Cheung J. W., Chung J. H. (2013). Adenosine-Induced Atrial Fibrillation: Insights into Mechanism. *Circulation: Arrhythmia and Electrophysiology*.

[29] Miyazaki S., Kobori A., Hocini M. (2013). Clinical Utility of adenosine-infusion Test at a Repeat Atrial Fibrillation Ablation Procedure. *Heart Rhythm*.

[30] Palamà Z., Nesti M., Robles A. G. (2022). Tailoring the Ablative Strategy for Atrial Fibrillation: a State-of-the-Art Review. *Cardiology Research and Practice*.

[31] Dukkipati S. R., Neuzil P., Kautzner J. (2012). The Durability of Pulmonary Vein Isolation Using the Visually Guided Laser Balloon Catheter: Multicenter Results of Pulmonary Vein Remapping Studies. *Heart Rhythm*.

[32] Zhao Y., Di Biase L., Trivedi C. (2016). Importance of Non-pulmonary Vein Triggers Ablation to Achieve long-term Freedom from Paroxysmal Atrial Fibrillation in Patients with Low Ejection Fraction. *Heart Rhythm*.

[33] Takamiya T., Nitta J., Inaba O. (2021). Impact of diagnosis-to-ablation Time on Non-pulmonary Vein Triggers and Ablation Outcomes in Persistent Atrial Fibrillation. *Journal of Cardiovascular Electrophysiology*.

[34] Morita H., Zipes D. P., Morita S. T., Wu J. (2012). The Role of Coronary Sinus Musculature in the Induction of Atrial Fibrillation. *Heart Rhythm*.

[35] Arruda M., Mlcochova H., Prasad S. K. (2007). Electrical Isolation of the Superior Vena Cava: an Adjunctive Strategy to Pulmonary Vein Antrum Isolation Improving the Outcome of AF Ablation. *Journal of Cardiovascular Electrophysiology*.

[36] Hsu L. F., Jaïs P., Keane D. (2004). Atrial Fibrillation Originating from Persistent Left Superior Vena Cava. *Circulation*.

[37] Valderrábano M., Chen H. R., Sidhu J., Rao L., Ling Y., Khoury D. S. (2009). Retrograde Ethanol Infusion in the Vein of Marshall: Regional Left Atrial Ablation, Vagal Denervation and Feasibility in Humans. *Circulation: Arrhythmia and Electrophysiology*.

